# Neutrophil-to-Lymphocyte Ratio Is a Potential Prognostic Biomarker in Patients with Ovarian Cancer: A Meta-Analysis

**DOI:** 10.1155/2017/7943467

**Published:** 2017-07-26

**Authors:** Shubo Chen, Liu Zhang, Guangyue Yan, Sijin Cheng, Abdel Hamid Fathy, Nana Yan, Yongzhao Zhao

**Affiliations:** ^1^Department of Oncology, Wuxi Second Hospital, Nanjing Medical University, Wuxi, China; ^2^School of Medicine, Tongji University, Shanghai, China; ^3^Department of Endocrinology, Hebei General Hospital, Shijiazhuang, China

## Abstract

**Background and Aims:**

Plenty of studies were conducted to explore the prognostic significance of neutrophil-to-lymphocyte ratio (NLR) in ovarian cancer with contradictory results. This study aims to summarize the prognostic significance of NLR in patients with ovarian cancer.

**Methods:**

A literature search in PubMed, Cochrane Library, and Embase was conducted. The endpoints were progression-free survival (PFS) and overall survival (OS).

**Results:**

Eleven studies involving a total of 2,892 patients were identified. The results indicated that patients with high NLR had shorter PFS compared to patients with low NLR in ovarian cancer (HR = 1.55, 95% CI = 1.15–2.08, *p* = 0.004, and *I*^2^ = 61%). Similarly, high NLR was related to shorter OS (HR = 1.51, 95% CI = 1.03–2.23, *p* = 0.04, and *I*^2^ = 85%). Moreover, high NLR was significantly associated with shorter PFS when the NLR cut-off was less than 3.3 (*p* = 0.03) or when treatment is operation (*p* = 0.002). In addition, high NLR was distinctly related to worse OS in Asian people (*p* = 0.04) or operation (*p* = 0.04).

**Conclusion:**

High NLR was associated with shorter PFS and shorter OS in ovarian cancer. NLR is potentially a promising prognostic biomarker in patients with ovarian cancer.

## 1. Introduction

Ovarian cancer is one of the most common gynecological diseases in oncology, and there were 238,700 estimated new cases and 151,900 related deaths in 2012 all over the world [1]. Lots of patients are at advanced stage at the first diagnosis, which accounts for the high mortality. The mainstream therapy for early ovarian cancer is still the operation with or without adjuvant chemotherapy, and chemotherapy is the most common therapy for the advanced ovarian cancer. However, the outcome of the primary therapy remains poor, around 50% of the patients will have a relapse occurring within 16 months, and the 5-year overall survival rate is still below 50% [2]. Therefore, more attention is paid to the promising prognostic factors to improve the prognosis of ovarian cancer, and the promising prognostic factors mainly include age, stage, and tumor biomarkers [3–5].

As is well-known, inflammation plays an important role in tumor growth, invasion, and metastasis. And the prognostic value of systemic inflammatory response (SIR) markers has been well studied, which included platelet to lymphocyte ratio (PLR), C-reactive protein, and MicroRNAs [6, 7]. In recent studies, neutrophil-to-lymphocyte ratio (NLR) was identified as a crucial prognostic biomarker in various tumors [8–13]. Meanwhile, plenty of studies were carried out to explore the prognostic significance of neutrophil-to-lymphocyte ratio in ovarian cancer; however, the results were contradictory [14–24]. The study conducted by Thavaramara et al. 2011 presented that there were no obvious relationship between the NLR and PFS (≤2.60 versus >2.60) (HR = 0.70, 95% CI = 0.35–1.40, and* p* = 0.344) or OS (≤2.60 versus >2.60) (HR = 0.70, 95% CI = 0.31–1.60, and* p* = 0.399) [16], and similar results were detected in the study conducted by Asher et al. in terms of OS (≤4.0 versus >4.0) (HR = 0.87, 95% CI = 0.52–1.44, and* p* = 0.575) [15]. Besides, Miao et al. covered that high NLR was associated with worse PFS (≤3.02 versus >3.02) (HR = 1.73, 95% CI = 1.23–2.45, and* p* = 0.002) or OS (≤3.02 versus >3.02) (HR = 1.62, 95% CI = 1.14–2.29, and* p* = 0.007) [22]; however, Zhang et al. reported that low NLR was an unfavourable factor in terms of PFS (≤3.40 versus >3.40) (HR = 0.50, 95% CI = 0.36–0.68, and *p* < 0.001) and OS (≤3.40 versus >3.40) (HR = 0.46, 95% CI = 0.33–0.65, and *p* < 0.001) [19]. Hence, controversy focusing on the relationship between the NLR and prognosis of ovarian cancer indeed exists. The aim of this meta-analysis was to explore the prognostic significance of NLR in ovarian cancer.

## 2. Methods

### 2.1. Literature Search Strategy

A complete systematic literature search method was implemented using PubMed, the Cochrane Library, and Embase up to December 25, 2016. The search strategy was “((Ovarian cancer) OR (Ovarian Neoplasm) OR (Ovary Neoplasm) OR (Ovary Cancer) OR (Cancer of Ovary) OR (Cancer of the Ovary)) AND ((neutrophil-lymphocyte ratio) OR (neutrophil-to-lymphocyte ratio) OR (neutrophil AND lymphocyte) OR NLR)”. The reference lists were also checked. The irrelevant articles were excluded by scanning the titles or abstracts. The remaining articles were then reviewed comprehensively by reading the full text.

### 2.2. Inclusion Criteria

The included study should meet all the criteria as follows: (1) retrospective or prospective studies; (2) focusing on the role of NLR on the prognosis in ovarian cancer; (3) enough data to get the hazard ratio (HR) for progression-free survival (PFS) or overall survival (OS), along with their 95% confidence intervals (CIs) or* p* values; (4) published in English.

### 2.3. Data Extraction and Quality Assessment

All the manuscripts were independently reviewed by two investigators. The following data was abstracted: family name of the first author, year of publication, country of the study, ethnicity of the study, sample size, cut-off value of NLR, therapy, and survival analysis. The HRs of PFS or OS obtained directly or indirectly from published articles were integrated in the meta-analysis according to the study conducted by Tierney et al. [25]. The HR assessed with multivariate analysis was abstracted when the multivariate analysis and univariate analysis were both applied. Newcastle-Ottawa Quality Assessment Scale (NOS) was used to assess the quality of each study. Any discrepancies were discussed with the third investigator.

### 2.4. Statistical Analysis

All the meta-analyses were carried out by Review Manager Version 5.3 software. The prognosis outcomes were explored using the HR, along with the corresponding 95% CI. The prognosis outcomes contained the PFS or OS. The heterogeneity was assessed by Cochran's *Q* test and Higgins *I*^2^ across studies. The heterogeneity should be considered when *p* < 0.05 and/or *I*^2^ > 50%, and the random-effect model was used; otherwise, the fixed-effects model was used. Besides, Egger's test and Begg's test were both conducted to evaluate publication bias by Stata 12.0. The sensitivity analysis was conducted by Stata 12.0 to access the robustness of the results. *p* < 0.05 was considered statistically significant.

## 3. Results

### 3.1. Literature Search

**Figure 1:**
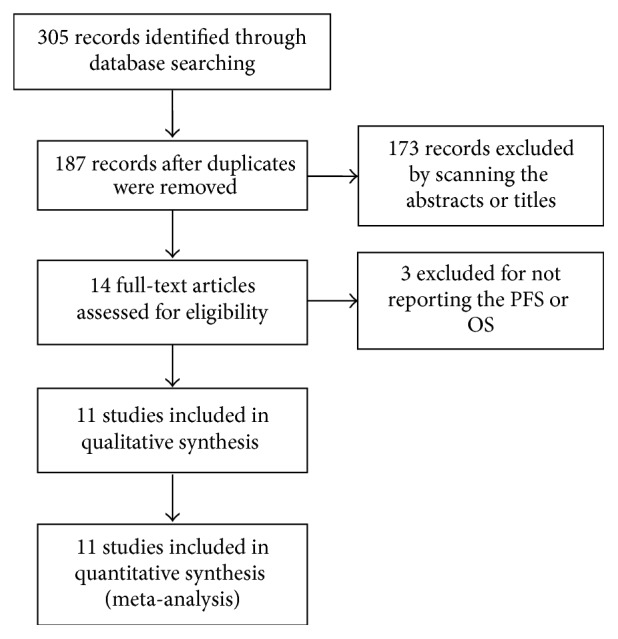
Flow diagram of study selection process.

As shown in Figure 1, a total of 305 papers retrieved, 187 papers remained after duplicates removed, and 173 papers were excluded by scanning the titles or abstracts. For the 14 potentially related studies remaining, 3 were excluded for insufficient datum to assess the prognosis outcomes. At last, 11 studies involving 2,892 patients were eligible for this meta-analysis [14–24].

### 3.2. Characteristics of Included Studies

As listed in Table 1, the eleven included studies contained 2,892 patients. Four studies paid attention to the role of NLR on the prognosis of patients receiving the chemotherapy [18, 19, 22, 23] and the others focused on the operation [14–17, 20, 21, 24]. The sample size varied from 30 patients to 875 patients. Besides, nine studies focused on the Asian [14, 16, 18–24] and two studies focused on the Caucasian [15, 17]. All the included studies reported the OS [14–24]; however, only seven studies covered the PFS [16, 18–22, 24]. In addition, nine studies reported the value of cut-off [14–16, 19–24]. As shown in Supplementary Table 1 (Supplementary Material, available online at https://doi.org/10.1155/2017/7943467), the main adjusted factors in the OS included age, stage, grade, PLR, CA125, and residual disease.

### 3.3. Meta-Analysis of PFS

**Figure 2:**
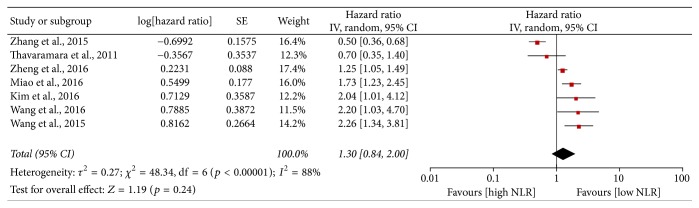
Meta-analysis of progression-free survival.

**Figure 3:**
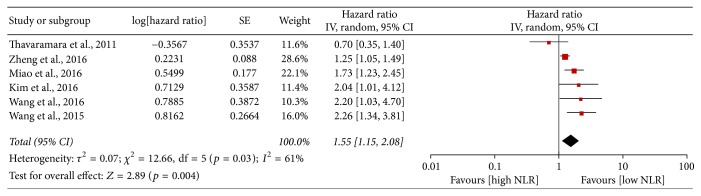
Meta-analysis of progression-free survival when Zhang et al. was excluded.

Eight studies reported the PFS; however, the study conducted by Cho et al. was excluded for data deficiencies [14]. As shown in Figure 2, no significant correlation was observed between the high NLR and PFS (HR = 1.30, 95% CI = 0.84–2.00, and* p* = 0.24), with large heterogeneity (*I*^2^ = 88%, *p* < 0.0001). Based on the sensitivity analysis conducted by Stata 12.0, Zhang et al. was finally excluded (Supplementary Figure 1). Therefore, six studies involving 1,726 patients were finally included in the meta-analysis of PFS and the HR of each study was assessed by multivariate analysis. As shown in Figure 3, in consideration of the large heterogeneity (*I*^2^ = 61%,* p* = 0.03), the random-effect model was employed. The results indicated that high NLR appeared to be a stronger predictor of risk when compared to low NLR (HR = 1.55, 95% CI = 1.15–2.08, and* p* = 0.004). Besides, sensitivity analysis demonstrated that the combined HRs of PFS did not significantly alter when excluding any study by turn (Supplementary Figure 2). And no publication bias among six included studies was detected (Begg test,* p* = 1.000; Egger test,* p* = 0.361).

Seven studies were enrolled into the subgroup analysis of PFS. As listed in Table 2, the subgroup analyses were carried out to investigate the sources of heterogeneity. Subgroup analysis stratified by NLR cut-off value showed that the high NLR was a risk factor when cut-off value <3.3 (HR = 1.39, 95% CI = 1.03–1.89, and* p* = 0.03;* I*^2^ = 53%), but no significant correlation between the NLR and PFS was observed when cut-off value ≥3.3. With respect to the therapy, the pooled effect estimates indicated a significant correlation between high pretreatment NLR and operation (HR = 1.29, 95% CI = 1.10–1.51, and* p* = 0.002;* I*^2^ = 47%), but no statistical significance between high pretreatment NLR and chemotherapy was detected.

### 3.4. Meta-Analysis of OS

**Figure 4:**
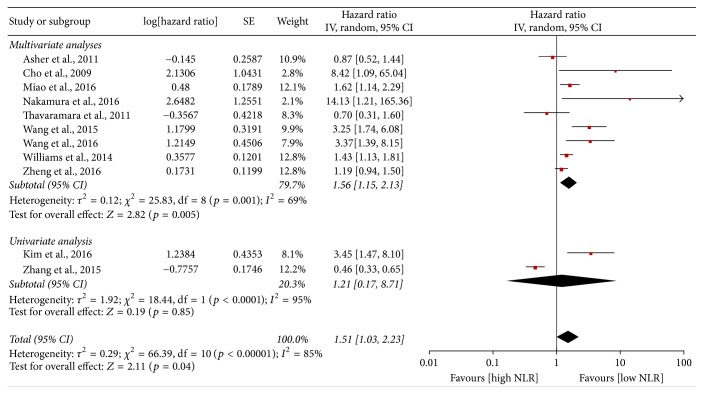
Meta-analysis of overall survival.

Eleven studies covered the OS and were included into the meta-analysis of OS. As shown in Figure 4, in view of the large heterogeneity, the random-effect model was applied (*I*^2^ = 85%, *p* < 0.0001). The meta-analysis revealed that patients with high NLR might have shorter OS compared to the patients with low NLR (HR = 1.51, 95% CI = 1.03–2.23, and* p* = 0.04). Regarding the subgroup analysis of multivariate analyses, the meta-analysis containing nine studies indicated significant superiority of a low NLR in ovarian cancer (HR = 1.56, 95% CI = 1.15–2.13, and* p* = 0.005), however, with clear heterogeneity (*I*^2^ = 69%,* p* = 0.0001). Meanwhile, the sensitivity analysis demonstrated that the combined HRs of PFS did not clearly alter when excluding any study by turn (Supplementary Figure 3). There was no publication bias among nine included studies (Begg test,* p* = 0.175; Egger test,* p* = 0.160). Nevertheless, in the subgroup analysis of univariate analyses, no obvious difference was observed between the patients with high NLR and patients with low NLR (HR = 1.21, 95% CI = 0.17–8.17, and* p* = 0.85;* I*^2^ = 95%).

As listed in Table 2, the subgroup analysis was employed in terms of OS. No apparent correlation was detected between the NLR and OS regardless of the NLR cut-off value <3.3 or ≥3.3. Regarding the ethnicity, the results presented that the patients with high NLR faced a greater risk of death in Asian population, with heterogeneity (HR = 1.76, 95% CI = 1.03–3.00, and* p* = 0.04;* I*^2^ = 87%), but no evident correlation between the NLR and OS was found in Caucasian population. In respect to the therapy, apparent correlation between the preoperation NLR and OS was found, and high NLR might predict worse OS when compared to the low NLR (HR = 1.45, 95% CI = 1.02–2.04, and* p* = 0.04;* I*^2^ = 68%); nevertheless, no significant relationship between the NLR and chemotherapy was observed.

## 4. Discussion

Increasing evidence has indicated that inflammatory response might be involved in the occurrence and growth of various tumors [26–31]. And inflammation-related neutrophils and lymphocytes are also crucial to tumor growth, invasion, and metastasis. NLR, a promising prognostic factor, has been fully researched in many kinds of tumors [32–34]. However, the association between high NLR and various cancers remains complicated. The mechanism underlying the association between high NLR and poor outcomes in various cancers remains unclear [6, 35, 36]. Some tumor-promoting cytokines might play a critical role in the development of tumorigenesis, such as nuclear factor kB (NF-kB) and transducer and activator of transcription 3 (STAT3) [37]. These tumor-promoting cytokines could change the expression level of cancer-related genes and promote normal cells to transform into cancer cells and then help the invasion and metastasis of tumor cells. In addition, with the assistance of cytokines, cancer cells might facilitate recruitment of tumor-associated neutrophils, which further help the tumor metastasis. Instead, lymphocytes are faithful anticancer defenders, and high lymphocyte counts have been proved as a favourable factor in terms of survival in a good way in many human cancers [38, 39]. The abovementioned mechanism might indicate that high NLR is an unfavourable factor in most cancers [28–31].

NLR is easily obtained from a routine blood test without additional cost. And the changes of NLR are breezily detected in the process of treatment of ovarian cancers. Therefore, NLR is a promising predictor in the individual treatment and more and more attention was paid to detecting the role of NLR on the prognosis of the ovarian cancer [14–24]. Several retrospective studies were carried out to determine the effect of NLR on the prognosis of the ovarian cancer, but with contradictory results [16, 18, 19, 21, 24].

In our meta-analysis, the results showed that high NLR was significantly associated with worse PFS when compared with the low NLR. And study conducted by Badora-Rybicka et al. also covered that pretreatment high NLR was a negative prognostic factor for ovarian cancer in terms of PFS [31]. However, Zhang et al. reported that high NLR was a favourable prognostic factor for ovarian cancer in terms of PFS (≤3.4 versus >3.4, HR = 2.012, 95% CI = 1.476–2.741, and *p* < 0.001) [19]. It is important to be noted that the study conducted by Zhang et al. only involved 190 patients and the HR of PFS was assessed by univariate analysis, which might heavily reduce the reliability [19]. Besides, high NLR was related to worse PFS when the value of cut-off <3.3 or before operation.

Regarding the OS, high NLR was significantly associated with worse OS, especially when the eligible studies were all assessed with multivariate analysis. Besides, the obvious correlation between the high NLR and poor OS was only observed in Asian, not in Caucasian, population. More studies in Caucasian people should be carried out because only two studies were enrolled into the subgroup analysis of Caucasian people. And Badora-Rybicka et al. also reported that high NLR was related to shorter OS compared with low NLR before the operation [31]. Nevertheless, Zhang et al. reported that high NLR was apparently associated with longer OS for ovarian cancer (≤3.4 versus >3.4, HR = 2.172, 95% CI = 1.545–3.054, and *p* < 0.001), which was assessed by multivariate analysis [19]. Besides, the heterogeneity of the meta-analysis of PFS obviously increased when Zhang et al. study was included into the analysis, which might heavily reduce the reliability. Therefore, Zhang et al. study was finally excluded from the current meta-analysis of PFS. In addition, the obvious correlation between the high NLR and shorter OS was observed for patients before operation.

The highlighted strengths of our meta-analysis are as follows. Firstly, at present, this study was the first meta-analysis to explore the prognostic significance of neutrophil-to-lymphocyte ratio in ovarian cancer. Secondly, eleven studies with a relatively large population were finally included; thus, the results were convincing. Thirdly, the subgroup analyses were carried out based on the NLR cut-off, therapy, and ethnicity; therefore, the analysis was comprehensive. However, some limitations of our study should be considered. Firstly, only one study reported the postoperative NLR on the prognosis of ovarian cancer and was not included into the study; therefore, the study only focused on the pretreatment NLR and on the prognosis of ovarian cancer. Secondly, plenty of analyses had a significant heterogeneity, which might reduce the accuracy of the results. Thirdly, the individual data was unavailable, like drug dose, curative time, and so on. Fourthly, some included studies reported limited information of the therapy; therefore, possible misclassification of therapy might exist in the current meta-analysis.

In summary, our study demonstrated that high NLR predicted worse PFS and OS in patients with ovarian cancer, especially significantly associated with shorter PFS when cut-off <3.3 or preoperation, and obviously related to worse OS in Asian people or preoperation. However, the conclusion should be used with caution for the limitations listed above and more multicenter prospective cohorts should be carried out to explore the prognostic significance of neutrophil-to-lymphocyte ratio in ovarian cancer.

## Supplementary Material

Supplementary Table 1: the main adjusted factors in the overall survival with multivariable analysis. Supplementary Figure 1: influence analysis of progression free survival excluding study conducted by Cho et al. Supplementary Figure 2: sensitivity analysis of progression free survival excluding two studies conducted by Cho et al and by Zhang et al respectively. Supplementary Figure 3: sensitivity analysis of overall survival assessed with multivariate analysis.

## Figures and Tables

**Table 1:** Characteristics of the included studies.

Study	Country	Ethnicity	Sample size	Cut-off	Therapy	Progression-free survival	Overall survival	NOS^†^
Cho et al. 2009 [14]	South Korea	Asian	192	2.6	Operation	NR^∫ ^	8.42 [1.09, 65.04]	7
Thavaramara et al. 2011 [16]	Thailand	Asian	129	2.6	Operation	0.70 [0.35, 1.40]	0.70 [0.31, 1.60]	6
Asher et al. 2011 [15]	United Kingdom	Caucasian	235	4.0	Operation	NR^∫ ^	0.87 [0.52, 1.44]	6
Williams et al. 2014 [17]	United States	Caucasian	519	NR^∫ ^	Operation	NR^∫ ^	1.43 [1.13, 1.81]	6
Wang et al. 2015 [18]	China	Asian	126	NR^∫ ^	Chemotherapy	2.26 [1.34, 3.81]	3.25 [1.74, 6.08]	8
Zhang et al. 2015 [19]	China	Asian	190	3.4	Chemotherapy	0.50 [0.36, 0.68]	0.46 [0.33, 0.65]	6
Miao et al. 2016 [22]	China	Asian	344	3.02	Chemotherapy	1.73 [1.23, 2.45]	1.62 [1.14, 2.29]	6
Kim et al. 2016 [21]	South Korea	Asian	109	2.8	Operation	2.04 [1.01, 4.12]	3.45 [1.47, 8.10]	6
Nakamura et al. 2016 [23]	Japan	Asian	30	3.91	Chemotherapy	NR^∫ ^	14.13 [1.21, 165.36]	6
Wang et al. 2016 [24]	China	Asian	143	3.43	Operation	2.20 [1.03, 4.70]	3.37 [1.39, 8.15]	6
Feng et al. 2016 [20]	China	Asian	875	3.24	Operation	1.25 [1.05, 1.49]	1.19 [0.94, 1.50]	8

^∫ ^NR, not reported; ^†^NOS, Newcastle-Ottawa Scale.

**Table 2:** Summary of the subgroup analysis results of NLR on PFS and OS.

Survival analysis	Included studies	Patients	HR 95% CI	*p*	*I* ^2^	*p* for heterogeneity
*PFS*						
Cut-off value						
<3.3	4	1,457	1.39 [1.03, 1.89]	0.03^‡^	53%	0.1
≥3.3	2	333	1.00 [0.23, 4.30]	1	92%	0.0004
Ethnicity						
Asian	7	1,916	1.31 [0.85, 2.03]	0.22	87%	<0.00001
Therapy						
Chemotherapy	3	660	1.23 [0.47, 3.24]	0.67	95%	<0.00001
Operation	4	1,256	1.29 [1.10, 1.51]	0.002^‡^	47%	0.13
*OS*						
Cut-off value						
<3.3	5	1,694	1.53 [0.99, 2.39]	0.06	67%	0.02
≥3.3	4	598	1.37 [0.51, 3.64]	0.53	88%	<0.0001
Ethnicity						
Asian	9	2,138	1.76 [1.03, 3.00]	0.04^‡^	87%	<0.00001
Caucasian	2	754	1.17 [0.72, 1.90]	0.52	68%	0.08
Therapy						
Chemotherapy	4	690	1.73 [0.60, 4.94]	0.31	93%	<0.00001
Operation	7	2,202	1.45 [1.02, 2.04]	0.04^‡^	68%	0.005

PFS, progression-free survival; OS, overall survival; ^‡^*p* < 0.05, the difference was significant.
