# Supplementary material for: Neutrophil-to-Lymphocyte Ratio Is a Potential Prognostic Biomarker in Patients with Ovarian Cancer: A Meta-Analysis
Source: Biomed Res Int. 2017 Jul 26;2017:7943467. doi: 10.1155/2017/7943467 (PMC5549495; doi:10.1155/2017/7943467)
Supplement: Supplementary file 1 — Supplementary Table 1: the main adjusted factors in the overall survival with multivariable analysis. Supplementary Figure 1: influence analysis of progression free survival excluding study conducted by Cho et al. Supplementary Figure 2: sensitivity analysis of progression free survival excluding two studies conducted by Cho et al and by Zhang et al respectively. Supplementary Figure 3: sensitivity analysis of overall survival assessed with multivariate analysis. [file 7943467.f1.docx]

**Supplementary Figure 1 Influence analysis of progression free survival excluding study conducted by *Cho et al*.**


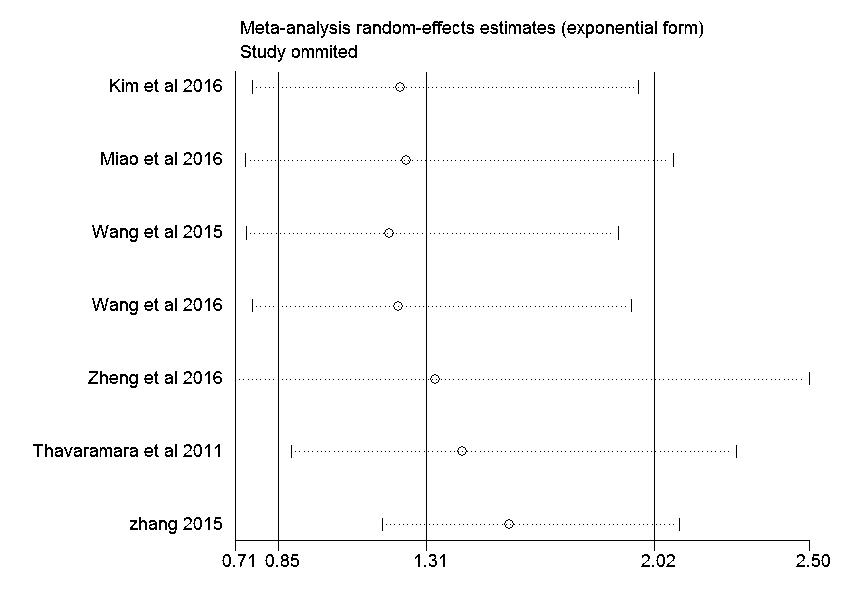


**Supplementary Figure 2 Sensitivity analysis of progression free survival excluding two studies conducted by *Cho et al* and by *Zhang et al* respectively.**


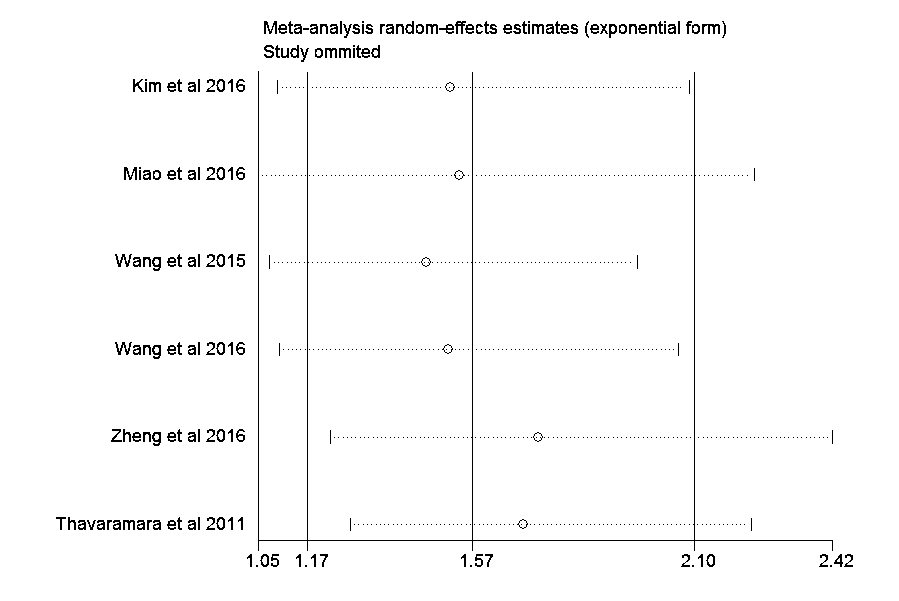


**Supplementary Figure 3 Sensitivity analysis of overall survival assessed with multivariate analysis**


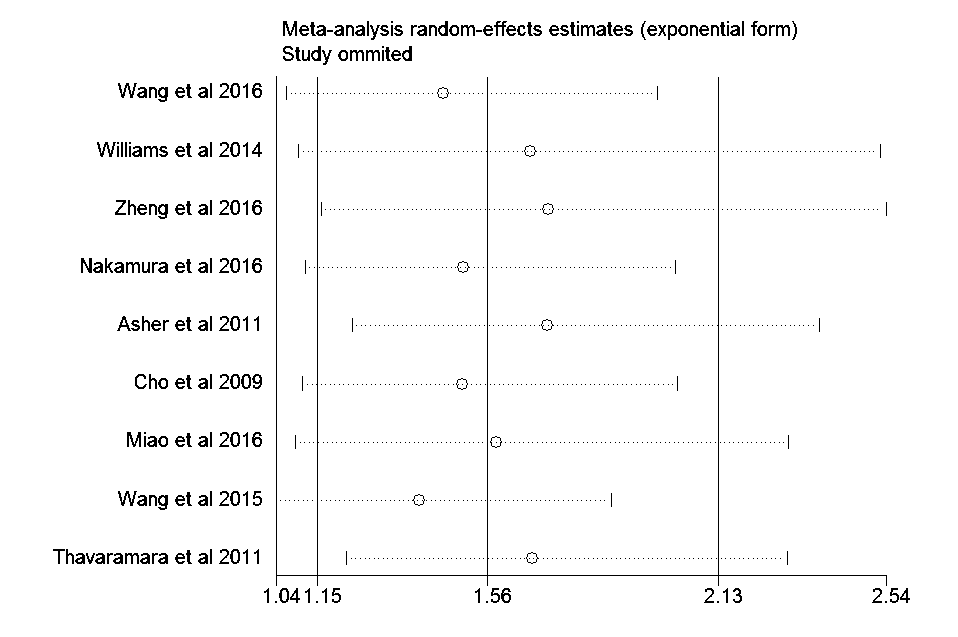


**Supplementary Table 1 The main adjusted factors in the overall survival with multivariable analysis**

| **Study** | **Age** | **Stage** | **Residual disease** | **Grade** | **PLR** | **CA125** | **Neutrophil** | **Malignant ascites** |
| --- | --- | --- | --- | --- | --- | --- | --- | --- |
| Cho et al 2009 [14] | √ | √ |  | √ |  | √ | √ |  |
| Thavaramara et al 2011 [16] |  | √ |  |  |  |  |  |  |
| Asher et al 2011 [15] | √ | √ | √ | √ | √ |  |  |  |
| Williams et al 2014 [17] |  |  |  |  |  |  |  |  |
| Wang et al 2015 [18] | √ | √ | √ |  | √ | √ | √ | √ |
| Miao et al 2016 [22] |  | √ | √ | √ | √ | √ |  | √ |
| Nakamura et al 2016 [23] |  |  |  | √ | √ |  |  |  |
| Wang et al 2016 [24] | √ |  |  | √ | √ | √ |  |  |
| Zheng et al 2016 [20] | √ | √ |  |  |  |  |  |  |

PLR= Platelet-to-lymphocyte ratio.
